# NSSC: a neuro-symbolic AI system for enhancing accuracy of named entity recognition and linking from oncologic clinical notes

**DOI:** 10.1007/s11517-024-03227-4

**Published:** 2024-11-01

**Authors:** Álvaro García-Barragán, Ahmad Sakor, Maria-Esther Vidal, Ernestina Menasalvas, Juan Cristobal Sanchez Gonzalez, Mariano Provencio, Víctor Robles

**Affiliations:** 1 Center of Biomedical Technology, Universidad Politécnica de Madrid, Campus Montegancedo, Pozuelo de Alarcón, 28223 Madrid Spain; 2 Data Science Institute, Leibniz University of Hannover, Welfengarten 1, Hannover, 30060 Lower Saxony Germany; 3 Scientific Data Management Group, TIB-Leibniz Information Centre for Science and Technology, Welfengarten 1B, Hannover, 30167 Lower Saxony Germany; 4 Oncology, Hospital Puerta de Hierro, Madrid, Spain

**Keywords:** Neuro-symbolic, LLM, NER, EL, EHR, Breast cancer

## Abstract

**Abstract:**

Accurate recognition and linking of oncologic entities in clinical notes is essential for extracting insights across cancer research, patient care, clinical decision-making, and treatment optimization. We present the Neuro-Symbolic System for Cancer (NSSC), a hybrid AI framework that integrates neurosymbolic methods with named entity recognition (NER) and entity linking (EL) to transform unstructured clinical notes into structured terms using medical vocabularies, with the Unified Medical Language System (UMLS) as a case study. NSSC was evaluated on a dataset of clinical notes from breast cancer patients, demonstrating significant improvements in the accuracy of both entity recognition and linking compared to state-of-the-art models. Specifically, NSSC achieved a 33% improvement over BioFalcon and a 58% improvement over scispaCy. By combining large language models (LLMs) with symbolic reasoning, NSSC improves the recognition and interoperability of oncologic entities, enabling seamless integration with existing biomedical knowledge. This approach marks a significant advancement in extracting meaningful information from clinical narratives, offering promising applications in cancer research and personalized patient care.

**Graphical abstract:**

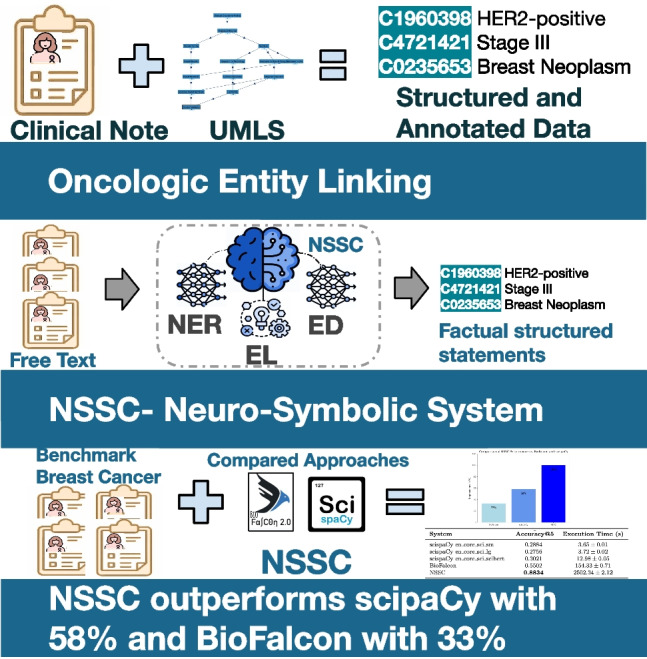

## Introduction

Cancer remains a major public health problem, ranking as the second leading cause of death worldwide. In particular, breast cancer is the most common cancer in women worldwide and the second most common cancer in general [1]. According to the World Health Organization,^1^ 2.26 million women worldwide will be diagnosed with breast cancer in 2022. Furthermore, cancer treatment is an expensive process with significant social and economic consequences for patients and healthcare systems [2].

Electronic health records (EHRs) encode relevant information about a patient, representing a rich source for supporting clinical research [3]. The oncology care process generates a large amount of information that describes the evolution of cancer in patients [4]. Physicians capture this information in the EHR using narrative clinical notes [5]. Extracting and mining this information is critical to supporting oncology research and improving patient outcomes. However, extracting this information is challenging due to the complexity of natural language. The use of natural language processing (NLP) in the biomedical domain has increased the possibility of automatically extracting information from clinical oncology narratives [6–8]. Recently, deep learning-based approaches have shown their feasibility in obtaining accurate information in the processing of clinical narratives on cancer [9–11].

In the Spanish language, several studies have also aimed to extract information in the cancer domain [12–14]. However, these approaches have focused only on recognizing medical entity recognition or on the detection of negations and uncertainties [15, 16] using separate processes. They also do not use methods for linking entities or structuring the information after it has been extracted. Another interesting reference in Spanish is the Cantemist Challenge (CANcer TExt Mining Shared Task) [17], presented during IberLEF 2020. This effort represents the first collective attempt to evaluate and improve the development of tools for named entity recognition, concept normalization, and clinical coding, specifically targeting cancer-related information in Spanish. However, this challenge focused only on a single entity, tumor morphology, and did not define a complete pipeline. Instead, it was divided into three completely independent phases: NER, NORM, and CODING.

**Fig. 1:**
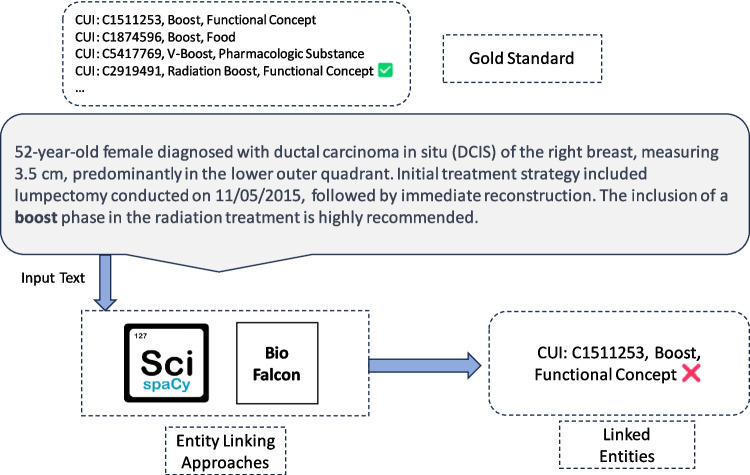
*Motivating example:* a clinical note snippet from a breast cancer patient, with correct UMLS terms as the gold standard (translated into English for clarity). Both scispaCy and BioFalcon fail to disambiguate and identify the correct terms, illustrating the need for contextual and semantic information to ensure accurate entity recognition in medical texts

The state-of-the-art NLP tools scispaCy [18] and BioFalcon [19] are designed to recognize medical entities and associate them with terms in controlled vocabularies (e.g., UMLS)—as illustrated in Fig. 1. However, these tools often overlook contextual information and rely solely on string similarity. Furthermore, while they can be applied to Spanish notes, there is no specific Spanish version of these tools.

In conclusion, the complexity of extracting valuable information from EHRs, especially in oncology, poses significant challenges due to the nature of clinical language. Existing approaches, especially in Spanish, either focus on isolated tasks such as entity recognition or do not implement entity linking or information structuring. In addition, state-of-the-art tools such as scispaCy and BioFalcon, while effective in English, lack tailored versions for Spanish clinical notes and rely primarily on string similarity.

**Problem statement** We address the problem of *oncologic entity linking* from medical notes, which involves accurately recognizing oncologic entities within the text and linking them to their corresponding terms in medical vocabularies.

**Proposed solution** We present a novel approach called the NeuroSymbolic System for Cancer (NSSC), a hybrid AI framework specifically designed to extract and organize oncology terms from clinical narratives. NSSC processes free-text clinical documents written in a narrative format, automatically identifies all relevant medical entities, and links them to the appropriate terms in the Unified Medical Language System (UMLS). To the best of our knowledge, NSSC is the first system capable of automatically structuring information from oncology clinical narratives, specifically for breast cancer, in Spanish.

Our approach integrates symbolic reasoning with language models to effectively recognize oncologic entities and link them to UMLS^2^ based on real breast cancer clinical notes. First, language models such as BERT [20] are used to exploit the contextual knowledge encoded in clinical notes during the NER step. This first step is already addressed in [21]. Next, a symbolic system is able to reason over a background knowledge that encodes relationships between medical concepts and their labels and semantic types in UMLS. Finally, large language models (LLMs) such as GPT-4 are used for their advanced natural language understanding capabilities. These models assist in disambiguating the most appropriate UMLS term(s) by evaluating and interpreting the context around each recognized medical entity, thereby selecting the most relevant term from a number of potential options.

We have conducted extensive validation experiments to assess the NSSC effectiveness. We have developed an in-house benchmark to compare NSSC with existing approaches. The validation process includes rigorous evaluation metrics to demonstrate the robustness and reliability of NSSC in handling different clinical scenarios.

Our work makes several significant contributions to the field of clinical natural language processing, particularly in the area of oncology:

- **First integrated system for Spanish-specific clinical narratives:** We present the first fully integrated system for extracting, linking, and structuring oncologic entities from Spanish-language clinical narratives. In doing so, we address the significant gap in the availability of NLP tools for Spanish medical texts.
- **Generalizable and cost-effective system:** Our approach is designed to be adaptable beyond breast cancer, making it versatile for other types of cancer and potentially other medical conditions. It is also cost-effective because it does not rely on resource-intensive pre-labeling of data.
- **Novel disambiguation method based on LLMs:** We propose a novel disambiguation method that exploits the capabilities of LLMs, such as GPT-4. This approach goes beyond traditional string similarity methods by using the contextual understanding power of LLMs to accurately identify and link the most appropriate UMLS terms, even in cases of ambiguous or complex medical terminology.

The rest of the paper is organized as follows: Section 2 summarizes the state of the art, and Section 3 presents the addressed problem and our proposed solution based on a hybrid AI system. Next, the NSSC algorithms are described in Section 4, and the results of the empirical evaluation are reported in Section 5. We discuss the observed results in Section 6, then the limitations are described in Section 7, and finally, we conclude with the conclusions in Section 8.

## Related work

Recent advances in hybrid AI systems have demonstrated significant improvements in various domains. The Neuro-Symbolic System for Cancer (NSSC), CapMatch [22], and Perceptive Capsule Network (PCapN) [23] exemplify this trend. NSSC integrates symbolic reasoning, LLMs, and deep learning for oncologic entity recognition, while CapMatch combines capsule networks, contrastive learning, and knowledge distillation for human activity recognition (HAR) from wearable sensors. Similarly, PCapN employs capsule networks and distillation methods for multivariate time series classification. Despite their different applications—NSSC in medical text, CapMatch in sensor data, and PCapN in time series—each system emphasizes advanced representation learning, knowledge transfer, and context-aware processing, demonstrating the versatility of hybrid AI approaches.

Building on these recent advances, this section reviews the state of the art in normalization of free text in biomedical data, focusing on three core areas: named entity recognition-NER (Section 2.1), entity linking-EL (Section 2.2), and named entity disambiguation-NED (Section 2.3). We analyze different approaches, including those based on deep learning, knowledge bases (KB), symbolic methods, and hybrid AI systems. We also highlight gaps in current approaches, setting the stage for further innovation (Section 2.4).

### Named entity recognition

Named entity recognition (NER) is a technique used to classify words in free text into predefined categories such as person names, locations, or medical terms. State-of-the-art NER techniques predominantly rely mainly on deep learning methods [24], incorporating input representations such as GloVe with BERT document-level embeddings [25], Cloze-style language model embeddings [26], or GloVe with aggregated contextual embeddings [27]. These inputs are processed by context encoders such as LSTM [25, 26] or RNN [27], and the outputs are decoded by tag decoders such as CRF [26, 27]. These approaches achieve high performance, with accuracy rates of up to 93.5% on the CoNLL-2003 dataset [26] and 92.07% on the OntoNotes5.0 dataset [25].

Although NER has traditionally focused on identifying entities without explicit linking, recent advances have integrated knowledge bases (KBs) to improve entity recognition accuracy by leveraging pre-existing entries. However, this approach is more naturally aligned with the entity linking process, as discussed in Section 2.2. NER methods based on KBs often have limitations compared to deep learning models, primarily due to their dependence on predefined databases, limited adaptability, and lack of contextual understanding. Conversely, deep learning models excel are characterized by scalability, contextual understanding, and automatic learning, resulting in more accurate and adaptive entity recognition. However, they require large annotated corpora to perform effectively, which can be a significant barrier in specialized domains, such as breast cancer.

There are specific cases where corpus annotation is unnecessary, especially when the text is very similar to entries in the KB. In such scenarios, KB methodologies that incorporate rule-based systems can be advantageous. For instance, [28] proposes a novel approach to train NER models without labeled data, achieving a seven-point improvement in F1 scores on the CoNLL 2003 and Reuters/Bloomberg datasets.

The use of NLP approaches to extract information from clinical texts has increased in recent years [29–31]. Specifically, within cancer-related clinical texts, models such as RoBERTa have demonstrated good performance, as shown by [32]. While deep learning methods achieve high accuracy and adaptability, they still struggle with domain-specific challenges, particularly when context is complex or the data is sparse. In contrast, NER methods using KBs lack the contextual understanding and adaptability of DL models but can be beneficial in specific scenarios where text closely matches KB entries. This highlights a gap in the development of more robust approaches that can handle unannotated data and specialized domains, such as clinical text, where models such as RoBERTa have shown promise. Future research should focus on bridging the gap between the scalability of deep learning methods and the accuracy of KB-based approaches while improving domain-specific NER capabilities.

### Entity linking

Entity linking (EL), also known as entity normalization, is the process of matching entities mentioned in a text with their corresponding entries in a KB. This step typically follows the extraction of entities from the text, where (entity, label) tuples are obtained. Given the complexity of natural language—including misspelling and inherent ambiguity— this information must then be normalized.

State-of-the-art EL models use neural architectures that have proven superior to classical ML methods [33]. These models can be categorized into four main types: joint mention detection and linking, global models, domain-independent approaches (including zero-shot methods), and cross-lingual techniques. Recent advances have shifted towards self-attention architectures such as BERT for mention encoding, with zero-shot methods becoming increasingly prevalent. Cross-encoder architectures, such as E-BERT [34] and BART-based models [35], have also demonstrated strong performance for joint tasks.

The EL task has seen applications using KBs, from graph traversal methods [36] to neural network-based approaches [37]. De Cao et al. [38] introduced a generative model that eliminates the need for hard negative sampling during training. These methods improve EL performance by integrating additional knowledge such as entity definitions, entity types, and knowledge graph (KG) triples to support training [39]. Despite these advances, models still rely heavily on a fixed number of candidates, approach the problem as a classification task, and require extensive training data. To mitigate the dependence on labeled datasets, some techniques [40] have begun to use unlabeled corpora. Recent efforts have focused on developing zero-shot models capable of generalizing EL to previously unseen entities [41, 42].

Symbolic approaches in artificial intelligence have also gained popularity [43]. Sakor et al. [19, 44] propose rule-based methods for linking entities in short texts using knowledge graphs such as DBpedia [45] and Wikidata [46]. These approaches highlight the importance of linguistic rules in understanding sentence structure, which helps in identifying the context identification of entities. Similarly, [47] presents a lightweight method for linking entities in Wikidata based on heuristic rules.

Hybrid AI approaches to EL, which combine symbolic reasoning with neural networks, are an active area of research aimed at improving the explainability of algorithms. Jiang et al. [48] introduced LNN-EL, a neuro-symbolic EL approach that combines human-defined rule templates with neural learning, providing interpretability and transferability without extensive labeled data. However, this method is primarily designed for short texts, such as questions, which limits its generalizability to more complex domains. Plu et al. [49] propose a hybrid method that combines a linguistic-based approach combined with annotation coverage facilitated by a KB.

In [50], they perform EL as an information retrieval (IR) task. However, in [51], they propose BertMCN, which uses BERT as a multi-classification task to normalize for mapping health-related entity mentions. In addition, [52] improves the model of Pattisapu et al. [53] for medical concept normalization by jointly learning the representations of the input and target concepts using RoBERTa as the embedding generator. This approach improves accuracy by up to 2.31% on three standard datasets. The improvement is significant because previous approaches only train the transformation of concepts into embeddings separately from the mentions that appear in the text. Recent work, such as Gallego et al. [54] (2024), proposed ClinLinker, which implements a two-stage pipeline using a SapBERT bi-encoder for candidate retrieval and a SapBERT cross-encoder for re-ranking, specifically tailored to handle Spanish medical texts using UMLS as KB.

The EL literature highlights gaps in generalizability across domains and scalability with large KBs. While zero-shot and cross-lingual methods, they still struggle with unseen entities and low-resource languages. Furthermore, the integration of symbolic and neural approaches is limited, with current models often failing to handle complex and ambiguous entities effectively. Future research should focus on developing more domain-agnostic models, improving efficiency with large KBs, improving zero-shot learning, and better integrating symbolic reasoning with neural methods to address these challenges.

### Named entity disambiguation

Named entity disambiguation (NED) is an NLP task that aims at resolving ambiguities arising from named entities in text. It can be considered as a subtask of EL, where NED is responsible for selecting the most appropriate candidate from a list generated for a mention in the text, based on context [55].

The NED task has been a subject of investigation for some time. For example, [56] proposed a method using a disambiguation SVM kernel trained on an online encyclopedia to detect and disambiguate named entities in open domain text. Novel approaches have been explored using large language models (LLMs) for disambiguating homonyms in academic KGs [57].

Symbolic approaches utilizing KBs have been a significant area of interest for NED tasks. For instance, [58] addresses spatial NED by identifying and assigning precise coordinates to ambiguous place names in text. Additionally, [59] focuses on applying NED to short text fragments in KGs, proposing an approach that includes context expansion with WordNet, coherence exploitation between entities, relation-based similarity calculation, and the use of syntactic features to improve the accuracy of query answering systems.

In [60], a technique for integrating cross-domain data by transferring structural knowledge from a general text KB to the medical field significantly improved disambiguation performance on benchmark datasets such as MedMentions and BC5CDR. Supervised classification approaches that identify informative keywords in the context of named entities have improved transferability [61]. Graph Neural Networks (GNNs) such as GraphSAGE, R-GCN, and MAGNN achieve state-of-the-art results by representing entities as query graphs and employing effective negative sampling strategies [62].

Recently, BELHD [63] introduced two major improvements: (i) the extension of homonyms in the KB with unique disambiguating strings, ensuring unambiguous linking decisions, and (ii) the implementation of a new strategy in contrastive learning that selects candidates more effectively, improving the training process. Further research [64] has also adopted NED, focusing on a retrieve-and-rerank approach.

Despite progress, the state of the art in NED reveals several research gaps and opportunities for further exploration. Traditional approaches using SVM and symbolic methods with KBs have made progress in disambiguating entities, especially in specific domains such as geography and short text fragments. However, these methods often struggle with complex or cross-domain scenarios where large LLMs and advanced neural methods, such as GNNs, show promise. There is still a need for more effective context handling, especially in domain-specific applications such as medical texts. Recent techniques, like contrastive learning and improved homonym handling, represent steps forward, but further research is needed to improve the integration of domain-specific knowledge with general-purpose models, to increase the portability of these approaches across different domains, and to develop more robust methods for handling ambiguous entities in different contexts.

### Summary and gaps in existing research

Several research gaps remain in NER, EL, and NED. Current deep learning models, while powerful, require large amounts of annotated data, which are often lacking in specialized domains such as oncology. Meanwhile, KB-based approaches lack the contextual adaptability of DL models. Future research should focus on developing more robust hybrid models that combine the contextual understanding of deep learning with the specificity and interpretability of KB-based approaches. Additionally, there is a need for models that can better handle low-resource languages and domain-specific nuances, particularly in the biomedical domain. To solve the problem of oncologic entity linking, three approaches can be considered:

- Symbolic AI systems: These are rule-based systems that use syntactic or lexicographic rules from a KB. They provide clear and understandable solution paths but struggle with variations in text mentions that differ from vocabulary labels, since concepts can be expressed in multiple ways, and the same word can have different meanings depending on the context.
- Sub-symbolic AI systems: These systems, often based on deep learning, require extensive annotated data. They typically outperform symbolic systems in most NLP tasks but lack interpretability and rely heavily on extensive training data.
- Hybrid systems: These systems combine symbolic and sub-symbolic approaches to exploit the strengths of both, aiming for high performance while maintaining some degree of interpretability.

Table 1 provides a selection of related articles. Most approaches focus only on individual steps such as NER or EL, with few proposing a complete pipeline. To the best of the author’s knowledge, no approach is specialized in the cancer domain or specifically tailored to Spanish texts. NSSC addresses the need for cost-effective solutions to extract insights from oncologic free-text records by introducing a hybrid AI system. This system operates without the need for extensive data annotation and utilizes an explainable decision-making process.

**Table 1:** Comparison of existing approaches for normalizing biomedical text

Article	Method type	NER	EL	ED	KB	Text type	Benchmarks
Baevski et al. [26]	BERT with a cloze-style objective training				–	General text	CoNLL-2003
Luo et al. [25]	BiLSTM + CRF				–	General text	CoNLL-2002, CoNLL-2003, Ontonotes 5.0 English datasets
Solarte et al. [32]	RoBERTa domain specific pretraining				–	Cancer-related narratives	In-house corpus
García-Barragán et al. [21]	BERT finetuning + string similarity within a diccionary				Custom dictionary	Cancer-related narratives	In-house corpus
Gallego et al. [54]	BERT Bi-encoder + Cross-encoder				SNOMED-CT	Medical notes	DisTEMIST, MedProcNER
Angell et al. [64]	Clustering-based Inference				UMLS	Biomedical text	MedMentions, BC5CDR
Sakor et al. [19]	Rule-based KG with BM25 as search engine				WikiData	general short text	LC-QuAD 2.0, Simple Question
Ji et al. [50]	BM25 + BERT				SNOMED-CT, MedDRA, MEDIC	Biomedical texts	ShARe/CLEF, NCBI, TAC2017ADR
Kalyan et al. [51]	BERT encoder + highway network				SNOMED-CT, MEDRA, AMT, SIDER	Health colloquial tweets	CADEC-MCN, TwADR-L
Pattisapu et al. [53]	RoBERTa target encoding				SNOMED-CT	Social media health texts	CADEC, PsyTAR, SMM4H 2017, SNOMED-CT Synonyms
Sung et al. [65]	BioBERT + MML				MeSH	Biomedical texts	NCBI, BC5CDR, TAC2017ADR
Vretinaris et al. [62]	BioBERT + GNN				UMLS	Biomedical texts	MDX, MIMIC-III, NCBI, ShARe, Bio CDR
Logeswaran et al. [66]	BM25 + BERT DAP				Wikia	Multiple domain	In-house corpus
Liu et al. [57]	ChatGPT				–	Science and technology text	In-house corpus
Lihu Chen et al. [67]	BiLSTM + CNN				SNOMED-CT, MedDRA	Biomedical texts	ShARe/CLEF, NCBI, ADR
Shuang Chen et al. [68]	BERT-Entity-Sim				–	General text	AIDA-CoNLL
Jiang et al. [48]	Neuro-symbolic: BERT + rule-based				DBpedia	General short texts QA	LC-QuAD, QALD-9, WebQSP
Plu et al. [49]	Hybrid: KNN + Linguistic-based				DBpedia	Literature texts	OKE challenge
NSSC (Ours)	Neuro-symbolic: BERT finetuning + rule-based + LLM				UMLS	Cancer-related narratives	In-house corpus [69]

This table compares named entity recognition (NER), entity linking (EL), and entity disambiguation (ED) methods across various approaches. Key characteristics analyzed include method type, use of NER, EL, ED, associated knowledge bases, types of text handled, and evaluation benchmarks. The table highlights strengths and limitations in each approach, helping to understand their applicability in biomedical data normalization

**Table 2:** Summary of notation

Symbol	Explanation
*T*	Short medical note
$\mathcal {I}$	Set of medical entities
$\mathcal {J}$	Set of medical terms from a controlled vocabulary $\mathcal {V}$
*h*	Set of pairs in $\mathcal {P}(\mathcal {I} \times \mathcal {J})$
$\delta (h,T)$	Function quantifying cost of recognizing entities in *T* and terms in $\mathcal {J}$ expressing their meaning
*e*	A medical entity in $\mathcal {I}$
$h^*$	Optimal set of pairs in $\mathcal {P}(\mathcal {I} \times \mathcal {J})$ that minimizes the cost $\delta (h^*,T)$
$\phi (h,T)$	Function quantifying cost of correctly recognizing medical entities
$\phi '((e,t),T)$	Function quantifying cost of correctly recognizing a medical entity *e* corresponding to term *t*
$\rho (h,T)$	Function quantifying cost of correctly linking medical entities
$\rho '((e,t),T)$	Function quantifying cost of correctly linking *e* to *t*
$\alpha (h,T)$	Function quantifying cost of performing a model for entity recognition and linking
$\alpha '((e,t),T)$	Function quantifying cost of processing the tasks of recognizing *e* and linking *e* to *t*
$\gamma (.)$	Function that assigns a short medical note *T* to a set of medical entities $\mathcal {I}$
$t_e$	Set of candidate medical terms for the medical entity *e*
$l_e$	Label representing the context of an entity *e* in medical note

## NSSC—problem statement and proposed solution

The Neuro-Symbolic System for Cancer (NSSC) is designed to provide structure and semantics to unstructured short medical notes, addressing the critical need for normalization of clinical notes within specific medical domains, such as oncology. The primary motivation for developing NSSC is the lack of robust tools and methodos capable of effectively transforming these unstructured texts into structured data, which is crucial for advancing clinical research, decision-making, and patient care.

Our approach formulates the problem as an optimization task, with the goal of maximizing the accuracy of medical entity recognition and linkage while minimizing the associated costs. NSSC utilizes a hybrid AI model that integrates three distinct paradigms: deep learning, symbolic reasoning, and generative AI, each of which contributes uniquely to NLP tasks. By orchestrating components based on these three paradigms, NSSC effectively recognizes medical entities and links them to standardized medical terminologies within controlled vocabularies, such as the UMLS. Furthermore, the modular design of NSSC ensures its generalizability across different medical domains beyond oncology.

A formal definition of the problem addressed by NSSC is provided in the following subsection, as well as a design pattern outlining the core components of the proposed framework. Table 2 summarizes the notation used in the specification of NSSC.

### Formal problem statement

Given a set $\mathcal {I}$ of medical entities, a set $\mathcal {J}$ of medical terms from a controlled medical vocabulary $\mathcal {V}$, and a short note *T*. The problem of *oncologic entity linking* is to recognize from *T*, a set $h^*$ of the correct medical entities in $\mathcal {I}$ and their links to terms in $\mathcal {J}$, while minimizing the cost of $\delta (h^*,T)$.

1 $$\begin{aligned} h^*= \underset{h \in \mathcal {P}(\mathcal {I} \times \mathcal {J}) }{\arg \min } \delta (h,T) \end{aligned}$$

- $h^*$ is the set of recognized medical entities and their links to medical terms in $\mathcal {V}$ that optimizes $\delta (.)$, i.e.,
  $$\begin{aligned} h^*=\{(e,t) \mid e \in \mathcal {I} \wedge ~ t \in \mathcal {J} \wedge ~ e \textit{ appears in T } \wedge \textit{ t is the medical term for e } \} \end{aligned}$$
- $\delta (h,T)$ is a utility function that quantifies the cost of correctly recognizing the medical entities in *T* and the terms in $\mathcal {J}$ that express the meaning of the linked entity in the controlled vocabulary $\mathcal {V}$.
  $$\begin{aligned} \delta (h,T)= \phi (h,T) + \rho (h,T) + \alpha (h,T) \end{aligned}$$
  - Cost of correctly recognizing medical entities in *T*: $\phi (h,T)=\sum _{(e,t) \in h} \phi '((e,t),T)$:
    $$ \phi '((e,t),T)={\left\{ \begin{array}{ll} 0, &  \text {if { e} is a correct medical entity in { T}}\\ 1, &  \text {otherwise}; \end{array}\right. } $$
  - Cost of correctly linking medical entities in *T*: $\rho (h,T)=\sum _{(e,t) \in h} \rho '((e,t),T)$:
    $$\begin{aligned} \rho '((e,t),T)={\left\{ \begin{array}{ll} 0, &  \text {if { t} is the correct term for the medical entity { e} in { T}}\\ 1, &  \text {otherwise}; \end{array}\right. } \end{aligned}$$
  - Cost of executing a model for performing named entity recognition and linking: $\alpha (h,T)=\sum _{(e,t) \in h}$ $\alpha '((e,t),T)$, where $\alpha '((e,t),T)$ quantifies the cost of processing the tasks of recognizing *e* and linking *e* to *t*.

**Fig. 2:**
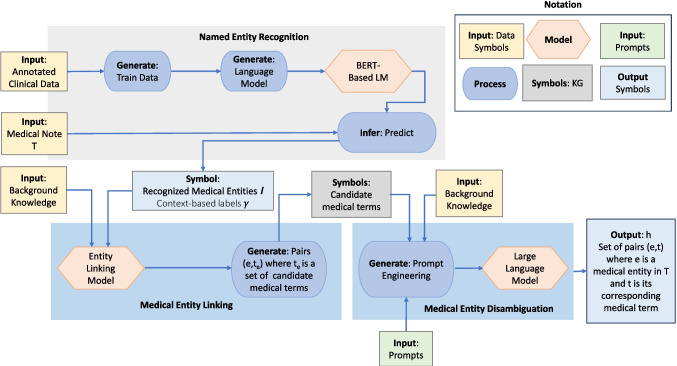
*Hybrid AI design:* hybrid design to recognize medical entities and their corresponding terms in medical controlled vocabulary such as UMLS

### Hybrid AI system: a boxology design pattern

NSSC is designed as a hybrid approach to recognize medical entities and to associate the recognized entities with medical terms in a controlled vocabulary. This hybrid approach combines a language model trained with annotated medical entities, a rule-based system guided by symbolic background knowledge, and a large language model to solve the optimization problem in Eq. 1.

The modular design pattern in Fig. 2 illustrates the usage of a hybrid AI system that combines these three AI subsystems. This *compositional* design pattern is based on the basic design patterns proposed by van Bekkum et al. [70] to describe hybrid AI systems. Thus, NSSC comprises the following components:

**Table 3:** NSSC configurable parameters

Parameter	Description
$q_{\text {type}}$	Specifies the format of the query to be executed against the BK to obtain the candidates concepts
$\theta _{\text {sim}}$	Threshold of the value of the similarity measure of the filtering module
$p_{LLM}$	The type of prompt used to query the LLM to disambiguation candidates

- **Named entity recognition (NER) component**: This component aims to identify oncologic entities within clinical text. Medical entities are recognized from medical notes based on a BERT-based language model pre-trained with oncologic terms from clinical notes. The NER component receives a clinical note *T* and produces a set of medical entities $\mathcal {I}$; for each recognized entity *e* in $\mathcal {I}$, it assigns a label $l_e$ representing its context in the clinical note. This annotation is captured in the function $\gamma (.)$ in a way that $\gamma (T)$=$\mathcal {I}$. Using a BERT-based NER procedure, the NER component systematically analyzes unstructured clinical notes in *T* to recognize relevant entities such as tumor types, treatment modalities, and relevant clinical concepts. This task of entity recognition task includes a contextual analysis mechanism to capture the broader context surrounding the identified oncologic entities. Contextual information is crucial for the disambiguation of entities. The NER component trains a model on a diverse dataset of clinical notes, ensuring the accurate extraction of entities along with their contextual information. The process to train this model is described in detail by García-Barragán et al. [21].
- **Medical entity linking (MEL) component**: A rule-based model is utilized to link medical entities to a list of candidate medical terms; a symbolic background knowledge serves as an extensional database to deduce candidate links. The MEL component receives the set $\mathcal {I}$ of medical entities recognized by the NER component and generates a set of pairs (*e*, $t_e$) where *e* belongs to $\mathcal {I}$ and $t_e$ is a set of candidate medical terms in UMLS. The MEL component resorts to a symbolic background knowledge (BK) to identify the potential terms from UMLS associated with *e*. The BK is built on top of the UMLS and represents medical terms using factual statements that associate the term with labels and semantic types. Since for the same medical term, different organizations may have assigned different labels to the term, BK keeps track of the number of duplicate labels and ranks the labels per term based on this number; we call this number the *linking score* of the labels of a term. Given an entity *e*, the MEL component searches on BK, the potential terms from UMLS that will be part of $t_e$. To increase confidence in the correctness of the terms in $t_e$, the MEL component follows a *heuristic-based* approach, which assumes that the labels with the highest values of the *linking score* are the most appropriate labels for *e*. Following techniques proposed by Sakor et al. [44], the MEL component indexes *e* and extends it with their synonyms. A BM25$^\dagger$ algorithm [71] is executed on the BK for querying and ranking entities in $\mathcal {I}$ based on *linking score*, including label, semantic type, score, and definition in each index. As a result, for each entity *e* in $\mathcal {I}$, a set $t_e$ of the top *k* candidates for links in UMLS is generated.
- **Medical entity disambiguation (MED) component**: Symbolic background knowledge is used for prompt engineering, and a set of prompts is generated to query a large language model (LLM) whose responses allow the disambiguation of the medical terms associated with each recognized entity. The generated prompts aim to maximize the efficiency of the large language model while minimizing the cost of using the model. The MED component receives triples $(e,l_e, t_e)$ and generates the set *h* corresponding to a solution of the optimization specified in Eq. 1. This component uses the predictive capabilities of LLMs and the reasoning process performed over BK to identify for each *e* and a singleton set $t_e$. The model captures the broader context surrounding the identified oncologic entities (provided as input), ensuring a holistic understanding of the entities and their interaction within the clinical notes, as well as the contextual information of the entity encoded in the BK. The medical entity linking process facilitates the extraction of CUIs based on the context and semantics of the identified entities, improving interoperability, consistency, and semantic alignment. This contextual information—collected from the background knowledge—is exploited for performing a Chain of Thought (CoT) [72] for engineering the prompts posed over the LLM.

## The NSSC algorithms

This section introduces the algorithms that implement NSSC, focusing on how they integrate symbolic reasoning with advanced language models to address the complexities of oncologic entity linking. By using a background knowledge base and a modular design, NSSC effectively handles the variations in medical terminology and context that are prevalent in clinical narratives. This ensures that terms such as “mama derecha” from the vocabulary are correctly recognized even when expressed differently, such as “mama parte derecha,” in clinical notes. The initial search generates a set of potential candidate matches. The algorithm then uses heuristics to determine whether these candidate terms should be passed on to an LLM for a more fine-grained disambiguation process. Actually, this mimics the process a human would use to associate a Spanish term with a medical vocabulary:

1. Recognize an entity in the text.
2. Search for the entity without any modifications.
3. Search for similar terms in the list of candidates.
4. If not found, translate the entity into English and perform the search again.

NSSC has three configurable parameters: the query type $q_{\text {type}}$, a threshold of the symbolic module $\theta _{\text {sim}}$, and a prompt $p_{LLM}$ to disambiguate concepts. Each parameter is described in Table 3.

The NSSC background knowledge (BK) has four specialized modules categorized by article [73]:

- Domain knowledge and ontology
  - *Indexer* stores and indexes vocabulary medical terms for retrieval.
  - *Semantic information* is used to store domain external information to make decisions about the disambiguation process.
- Trained models
  - *Clinical NER* corresponds to the trained model in the breast cancer domain for clinical entity recognition.
  - *LLM*: a generic model which is responsible for disambiguating candidates.

NSSC is presented in Section 4.1, which describes how a free text flow through the whole system. Each of the three components of our NSSC system is explained separately, entity recognition in Section 4.2, entity linking in Section 4.3, and entity disambiguation in Section 4.4. Finally, the algorithm complexity is discussed in Section 4.5.

### NSSC flowchart

**Fig. 3:**
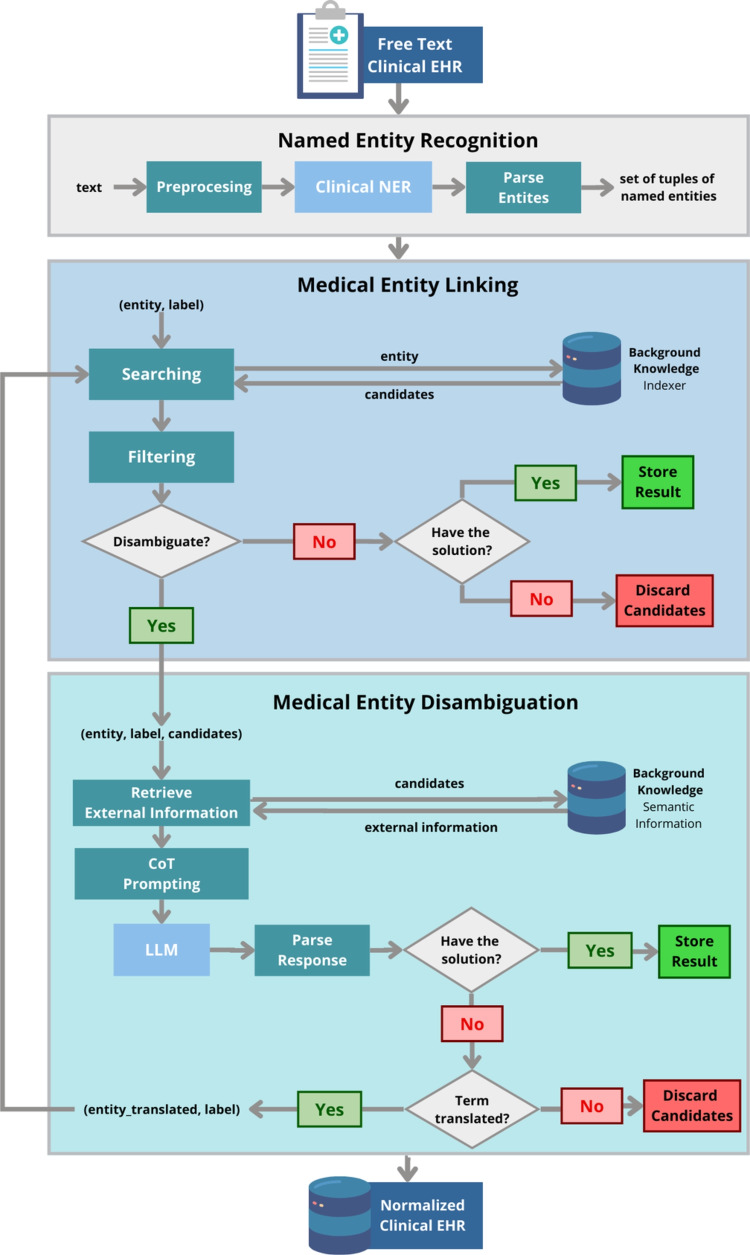
*NSSC flowchart:* clinical note *T* with the set $h^*$, which is a set of normalized terms aligned to UMLS. NSSC takes as input a short medical note in unstructured format and transforms this text into a set of medical entities. For each recognized entity, a background knowledge (BK) is searched to identify candidates for links in UMLS. In case disambiguation is required, semantic information from the candidate terms is used for Chain of Thoughts (CoT) prompting over an LLM

After training the models and configuring all parameters, NSCC normalizes new Spanish free text and extracts the corresponding CUIs. This online process is illustrated in Fig. 3. NSSC receives *T*, an oncologic clinical note which is initially passed to the NER module to obtain a set of entities $\mathcal {I}$, each represented as a tuple $(e, l_e)$. Subsequently, for each entity in $\mathcal {I}$, the medical entity linking module consults the BK using rule-based heuristics to make a decision. This decision determines whether we have an acceptable term *t* for an entity *e*, do not have it, or do not need to disambiguate. If disambiguation is required, the medical entity disambiguation module creates a prompt $p_{LLM}$ with the medical entity *e*, each label $l_e$, the candidate terms $t_e$, and their corresponding UMLS definitions. Ultimately, NSSC either successfully selects the best terms, finds them inadequate, or provides a translation of the term. If the term is translated, it is searched again, and the process is repeated.

For instance, given an input *T*, “Paciente diagnosticado con carcinoma ductal in situ,” the NSSC system will extract the set of entities $\mathcal {I} =$ {(“carcinoma,” “cancer concept”), (“ductal,” “cancer type”), (“in situ,” “cancer expansion”)}. Then, for each tuple, it assigns the most appropriate CUIs for each concept. For the concept “carcinoma,” the result might be (“carcinoma,” “Cancer Concept,” “{‘C0007097’}”), where “C0007097” is the CUI associated with the concept of “carcinoma.”

### Named entity recognition

NSSC uses a BERT-based model to perform NER. We choose for this technique because of the nature of natural language text, where the quality of written notes tends to be suboptimal due to the pressures faced by clinicians. Consequently, these models are essential because they adapt to language nuances and are able to identify words even with grammatical errors. These models require an annotated corpus to learn.

Algorithm 1 outlines the steps of the named entity recognition process. It receives a clinical note *T* and extracts a set of entities $\mathcal {I}$. This set contains tuples $(e, l_e)$, where $l_e$ is the medical label and *e* represents the text that appears in the prediction for a label $l_e$ by the NER model. This model is used after a preprocessing step. This step aims to prepare the clinical text before performing the information extraction task by splitting the clinical narrative into sentences and tokenizing each sentence. Acronyms are then transformed into full descriptions, such as by converting “ca.” to “cáncer.” This transformation is achieved using a combination of regular expressions and a dictionary of the most common acronyms in the cancer domain.

In a previous work [21], a model was trained to extract medical concepts using multilingual BERT [20]. NSSC utilizes this model, this approach that takes advantage of a transfer learning technique to perform clinical NER in the field of breast cancer. Transfer learning is achieved by fine-tuning the BERT model with a classification layer on top, as described in [16]. However, in [32], it is shown that RoBERTa generally outperforms BERT in medical text. In any case, the entity identification is a required step of the process that can be performed with the state-of-the-art methods that the researcher decides.

**Algorithm 1:**
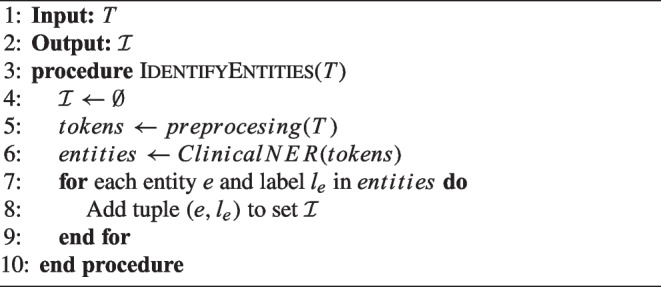
Named entity recognition.

### Medical entity linking

The NSSC medical entity linking uses a BK to generate the candidates; it is configured based on the following parameters:

- Background knowledge base: We have built a background knowledge [73] based on UMLS. For each medical entity, the background knowledge includes all its labels, semantic types, definitions, and synonyms in other knowledge bases, such as DBpedia or Wikidata. These descriptions provide contextual information collected by the communities contributing to UMLS and the knowledge bases.
- Search engine: The medical entity linking system resorts to OpenSearch^3^ to search for background knowledge. OpenSearch relies on indexes and search methods and is able to scale to large knowledge bases.
- Query to be posted to the search engine. As we will see, experiments have been conducted to find the most appropriate configuration of the search. The query may have the following configurable parameters:
  - *k*: It controls the number of candidates that the BK should return for a given medical entity. It is represented by a natural number.
  - *fuzziness*: The degree of fuzziness or tolerance in matching concepts.
    * Exact: This method is strictly an exact match. Only results that exactly match the query concepts in the BK.
    * Basic fuzzy: This method uses basic fuzzy matching, allowing for slight variations in the query terms.
    * Multi-match: This query method represents the highest level of fuzziness.
  - *lang*: The language of the terms is saved.
    * SPA: Spanish language.
    * ENG: English language.
  - *boost words*: Keywords that receive a boost in relevance.
- Similarity function in the optimization module: It determines the relationship between the vocabulary term and a medical entity.

Algorithm 2 is a sketch of the algorithm implemented for the medical entity linking system. When the results are retrieved from the search engine, they are ranked according to their relevance to the query. Each result is assigned a relevance score based on how well it matches the search query. With all of the results, we have a set of candidates that could be used directly as input to query the LLM. The result of the search will be a set of candidates ($t_e$) along with the ranking value.

**Algorithm 2:**
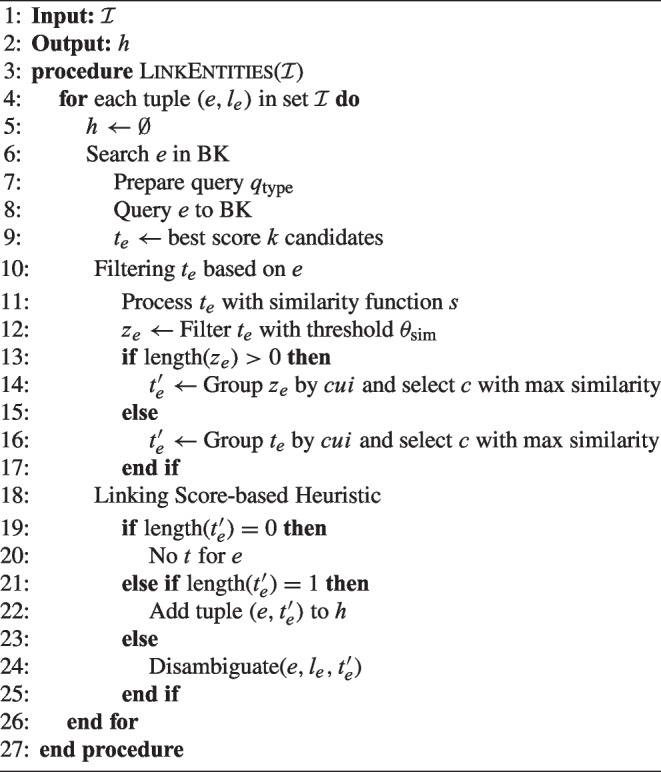
Medical entity linking.

The choice of query type is another customizable parameter within our system. Queries can be Exact, Basic_Fuzzy, Multi_match_SPA, Multi_match, and Multi_match_boosted. Details in Section 5.1.

#### Filtering

The results of searching in the knowledge base can be large. As we know, optimizing the number of tokens sent to an LLM is crucial for several reasons related to efficiency, computational cost, and model limitations. In particular,

1. Response time: Smaller inputs typically result in faster response times, which is critical for applications that require real-time or near real-time interactions.
2. Model output constraints: Some models have constraints on the maximum length of generated responses, and sending fewer tokens ensures that the response fits within those constraints.
3. Noise reduction: Sending extraneous or irrelevant tokens to the model can introduce noise and potentially affect the quality of the generated output.
4. Mitigate model bias: Expanded inputs can weaken the influence of essential details, potentially leading to biased or less accurate results.

As a consequence, our approach includes an optimization step in which the search results are grouped by *cui* and ranked with a matching function. We propose to use a similarity function *s*(.,.) that, when given a term of the candidate set (result of a search query in the search engine, $t_e$), returns a numeric value in the range of 0 to 1. Then, a threshold is defined so that only those candidates above the threshold are included in the final set of filtered candidates. This matching function is implemented in the Python documentation.^4^

2 $$\begin{aligned} s: \mathcal {I} \times \mathcal {J} \rightarrow [0,1] \end{aligned}$$

NSSC uses a threshold $\theta _{\text {sim}}$ that varies with context and requires configuration. The choice of this threshold for our case study is described in detail in Table 8.

#### Heuristic decision

At this stage, three different scenarios can unfold: (i) there are no candidates are in the set, indicating that the concept does not appear in background knowledge; (ii) the list contains only a single term; or (iii) the list encompasses multiple terms. In the second case, a direct mapping is established. In contrast, for the last scenario in our methodology, we utilize an LLM to facilitate the determination of the optimal solution, taking into account contextual nuances, the range of ranked candidates, and the definition of the concept in the specific vocabulary.

### Medical entity disambiguation

An LLM is used by NSSC to produce a result, considering the contextual information provided by the previous phase. This model offers insights into likely meanings based on the context in which the term is used. Creating a robust context is key to improving the performance of language models in disambiguation tasks. A detailed explanation of this process can be found in Algorithm 3. When using LLMs, several techniques can be used to control the behavior of the model and achieve the desired results. One important method is *prompt engineering*, which involves creating specific prompts to guide the model’s behavior. These prompts may include explicit instructions, contextual setting, or special formatting to guide the model towards the expected output. Two important *prompt engineering* techniques are as follows:

- Few-shot learning [74] involves giving the model a limited set of examples or demonstrations that illustrate the target behavior. This method helps the model better understand the intended task or concept more effectively. The model then uses these examples to generalize and formulate responses to novel prompts or questions, demonstrating understanding beyond the specific instances provided.
- Chain of Thought (CoT) [72] involves crafting prompts that encourage the model to reveal its reasoning process in a step-by-step manner, similar to how a human might think aloud while solving a problem. This approach is particularly effective for complex tasks, because it seeks not only the correct answer but also the logical path to that answer. By breaking down the thought process, CoT enhances the model’s ability to handle complex questions and provides users with a clear understanding of how the model reached its conclusions, thus improving transparency and trustworthiness.

Although NSSC is LLM-agnostic, as a proof of concept, we implemented this step using the OpenAI API.^5^ This API involves determining the appropriate model and includes configuration parameters such as the maximum number of tokens and the temperature of the language model. The optimal configuration of these parameters is essential to achieve the desired behavior.

In Section 5, the effects of combining different possibilities to assess their impact on the LLM’s outcome. Although the full text of the note in the prompt could offer the potential for richer interactions with the LLM, several challenges must be effectively addressed to make this approach successful.

- Computational costs: LLMs, especially those hosted in the cloud, can have computational costs associated with processing large amounts of text. The more tokens you send, the more resources are required for analysis.
- Response time: Sending lengthy prompts may result in longer response times from the LLM. Although this is not a real-time issue, it is something to consider.
- Privacy and sensitive information: Sending complete notes may involve sharing sensitive or private information with external language model services, which raises privacy concerns.
- Model capacity limits: Some language models have input limits and may have a maximum token limit per request, and sending very long requests may result in truncation or incomplete processing.

**Algorithm 3:**
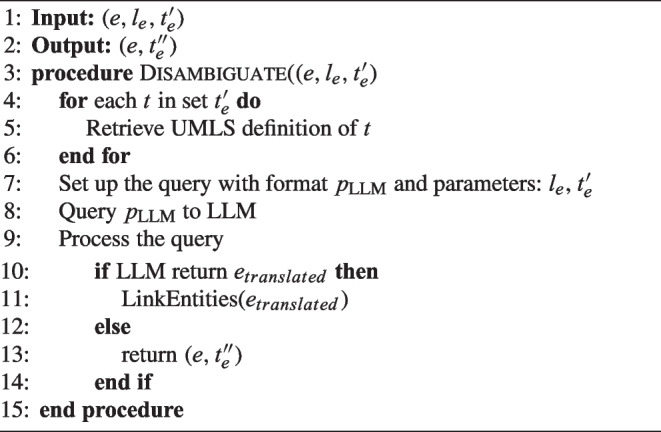
Medical entity disambiguation.

**Fig. 4:**
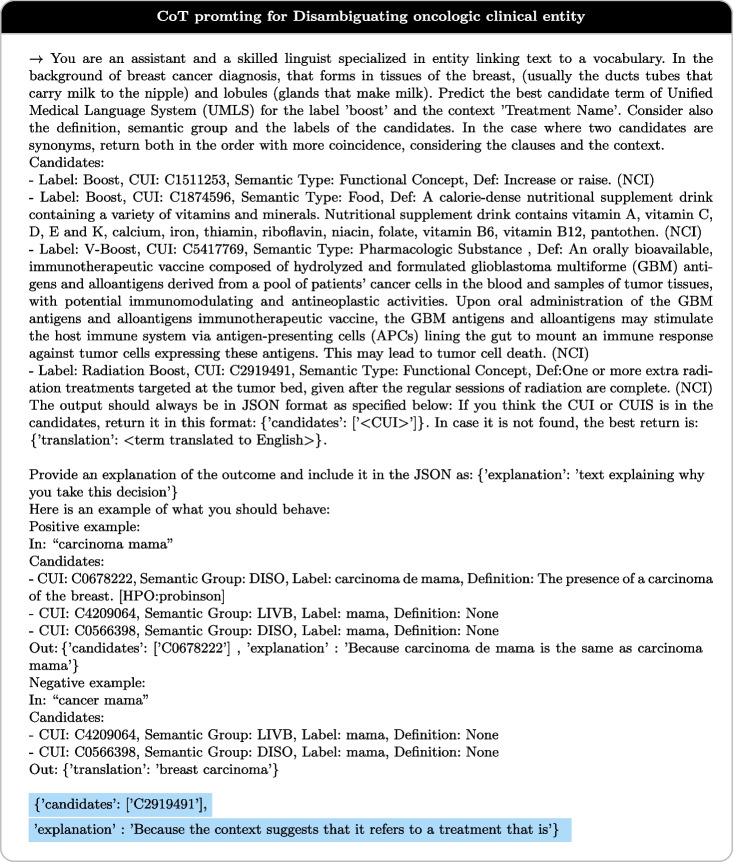
*Example for CoT prompt:* a prompt to disambiguate the entity “boost” and context “treatment name”

The best prompt for our approach is illustrated in Table 9, which is a CoT prompt. An example of CoT is illustrated in Fig. 4. In this example, we show how, by using the CoT prompt technique, the LLM can intelligently select the most appropriate CUI based on the contextual information provided. The language model is required to justify its decision by explaining whether a suitable CUI exists; if not, it should provide a translation of the term. In addition, each candidate is given the CUI, the label, the semantic group, and the definition of the CUI is provided. Because UMLS often provides multiple definitions for each CUI, we give preference to the definitions provided by the NCI (National Cancer Institute)^6^ because it is a recognized and authoritative source of cancer-related information.

### Algorithmic complexity

The time complexity of NSSC is determined by the sum of the time complexities of the three algorithms of which it is composed. Since NSSC takes a text input that is divided into an arbitrary number of tokens, the key parameters to consider for each module are as follows:

- NER: The BERT-based model must predict an entity for each token for a number of tokens. The preprocessing function time is minimal compared to the NER model.
  - $s$: Number of tokens is by divided the input text.
  - $b$: Number of neurons in the NER model.
- MEL: The BM25 algorithm has to compare all the terms in UMLS with the entities extracted from the previous step. Then, it has to filter the subset of candidates and decide whether to disambiguate or not.
  - $r$: Number of entities extracted from the text.
  - $u$: Number of terms in the UMLS.
  - $k$: Number of filtered candidates.
- MED: A prompt must be created for each entity that needs to be disambiguated. This prompt is then processed by the LLM, which generates a response token by token. For each new token, all the neurons in the model must be computed.
  - $l$: Number of neurons in the LLM.
  - $p$: Length of prompt, expressed in number of tokens.
  - $o$: Answer length generated by the LLM.

Given all these parameters, the time complexity of NSSC can be expressed as

$$\begin{aligned} O(NSSC)= &   O(NER) + O(MEL) + O(MED)\\= &   s \cdot b +( r \cdot u + r \cdot k ) + r \cdot p \cdot l \cdot o \end{aligned}$$

As is known, the disambiguation process is the most computationally expensive since the number of neurons in an LLM is about 100 billion. However, the NSSC algorithm is optimized to minimize the use of the LLM whenever possible. In the best case, the complexity of NSSC only involves the NER and MEL modules.

Table 4 summarizes the space complexity for the various components involved in the deployment, focusing on the required disk space.

**Table 4:** Space complexity of NSSC

Component	Size
SQL service (saves all UMLS definitions)	126 MB
OpenSearch service (saves all medical terms of UMLS)	2.3 GB
BERT-base NER Model	626 MB
LLM service	0 (API)
Code folder	6.6 GB
Total	**9.62 GB**

## Validation experiments

This section reports on the results of the experimental study of the NSSC performance. The aim is to assess NSSC and compare it with existing approaches. We use a case study in breast cancer with real clinical notes. The experimental study is guided by the following research questions (RQs):

**RQ1:** How does the integration of background knowledge through different query types affects the overall performance and effectiveness of NSSC?

**RQ2:** How does the cost-effectiveness of utilizing NSSC models for natural language processing tasks vary when employing different prompt techniques, taking into account the influence of token length on accuracy?

**RQ3:** How can the combination of LLMs and the background knowledge contribute to better disambiguation of entity linking, particularly in cases where multiple entities share similar or ambiguous surface forms within clinical notes?

The experimental setup is as follows:

**Dataset**. We have generated an in-house corpus containing clinical notes from nearly 600 breast cancer patients. All the notes correspond to the category of clinical judgement. The corpus has been annotated following the process described in [21] resulting in a total of 17,986 entities. Table 5 reports on the labels that have been used in the annotation process, their support in the corpus, and the number of tokens. The former is calculated using the spaCy^7^ tokenizer.

**Fig. 5:**
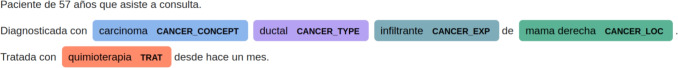
Annotations with breast cancer information

Figure 5 illustrates an annotated clinical note from our NER corpus. The phrase “Diagnosticada con carcinoma ductal infiltrante de mama derecha” contains four entities: cancer concept, cancer type, cancer expansion, and cancer location. It is important to note that identifying these named entities is only the first step. Subsequently, these medical entities are normalized to the UMLS vocabulary.

In order to validate the NSSC, we introduce an in-house validation benchmark, called the *Breast-Norm-Benchmark*, which is constructed from the NER corpus described above. This benchmark includes each term from the NER corpus only once, meaning that duplicate terms are not repeated. This benchmark is described in Table 6 and consists of 438 unique terms. These terms, along with their associated entities, will be the context provided to the LLM.

Below are two examples from the *Breast-Norm-Benchmark*:

- **Text:** “cuadrante superior externo mama izquierda” **Context:** “Cancer Location” **CUIs:** “{‘C1266903’}”
- **Text:** “mastectomía ahorradora” **Context:** “Surgery” **CUIs:** “{‘C0024881’, ‘C1997268’}”

During medical entity linking, we encounter the challenge that many UMLS terms are available only in English and not in Spanish. In our background knowledge—created using UMLS 2023AB–$-$25.8% of the terms are in English, while only 10.8% are in Spanish. To overcome this issue, we first search for the term in Spanish. If it is not found, we use the LLM to translate the term into English and then search for its corresponding English term in UMLS.

**Table 5:** Support of the annotated labels of the corpus

Label ($l_e$)	Support	#Tokens
Cancer concept	826	845
Cancer type	558	578
Cancer subtype	75	102
Cancer expansion	650	702
Cancer location	1645	2809
Cancer metastasis	369	388
Cancer recurrence	44	44
Molecular marker	1691	4346
Cancer stage	344	694
TNM	594	2176
Treatment name	351	444
Treatment schema	96	104
Treatment drug	475	487
Treatment frequency	43	54
Treatment quantity	56	101
Surgery	888	1259
Total	**8600**	**15,031**

**Implementation** NSSC is implemented in Python 3.10. The source code is located in https://github.com/SDM-TIB/NSSC. The experiments are executed in an Intel Core i9-7900X CPU with a clock speed of 3.30 GHz, equipped with 20 CPU cores, organized into 10 cores per socket, each supporting 2 threads per core. The CPU architecture was identified as x86_64. The RAM consisted of 4 x 16GiB DDR4 synchronous modules, each running at 2133 MHz. The O.S. is Linux with kernel version 5.15.0-83-generic, based on Ubuntu 20.04.1 LTS.

**Metrics** The performance of NSSC is measured in terms of accuracy (3). We use this metric because an entity can have more than one valid CUI in UMLS (e.g., the entity “chemotherapy” can be valid with the following CUIs: “C0013216,” “C3665472,” “C1571591”). This metric focuses on the proportion of correct (gold) values that the system also identified.

3 $$\begin{aligned} \text {{Accuracy}} = \frac{{\left| G \cap R \right| }}{{\left| G \right| }} \end{aligned}$$

where:

- *G* = set of gold (correct) values
- *R* = set of outputs by NSSC.

Furthermore, we use Accuracy@N (Eq. 4) to allow comparison with other systems, as classical methods cannot retrieve the best CUIs and are limited to providing a top-N set of results.

4 $$\begin{aligned} \text {{Accuracy@N}} = \frac{{\left| G \cap R_{\text {{top-N}}} \right| }}{{\left| G \right| }} \end{aligned}$$

where:

- $R_{\text {{top-N}}}$ includes the top N responses in the system’s output.

*Execution time* corresponds to the elapsed time spent to execute an NLP tool to solve the problem of *oncologic entity linking* in the entire benchmark. The execution time is measured using the Python library time. It is acknowledged that the timing of a general-purpose computer is influenced by the operating system scheduler. As a result, the reported times represent an average of five different runs conducted at various times. Finally, the metric *tokens(.)* quantifies the number of tokens generated by an LLM. In our experiments, *tokens(.)* is calculated using the OpenAI API.

**Table 6:** Validation benchmark

Context ($l_e$)	Support	#Tokens
Cancer concept	25	29
Cancer type	18	19
Cancer subtype	19	34
Cancer expansion	14	18
Cancer location	151	333
Surgery	69	138
Treatment drug	96	103
Treatment name	46	68
Total	**438**	**742**

**Table 7:** Performance for different queries to the search engine of BK

BK searching query	$\bar{\text {Accuracy@1}}$	$\bar{\text {Accuracy@15}}$	Execution time (s)
Exact	0.4276	0.5239	**1**.**72**
Basic_Fuzzy	0.3586	0.4184	19.37
Multi_match_SPA	0.3586	0.4184	92.51
Multi_match	0.4667	0.6051	84.71
Multi_match_boosted	**0**.**5402**	**0**.**7764**	137.46

Each method has its unique approach to querying, ranging from highly flexible (fuzzy) to precise (exact) matching

**Baselines** The following NLP tools are considered as baselines:

- *scispaCy* [18] is a specialized approach designed for processing biomedical and scientific texts, based on the spaCy library’s robust framework. It specifically addresses the complex requirements of biomedical information, providing an extensive array of tools and models designed for functions like named entity recognition, disambiguation, among others.
- *BioFalcon* [19] is an entity recognition and linking engine. It extends the background knowledge and the target KG of the Falcon approach from DBpedia and Wikidata to UMLS to support entity linking to UMLS. BioFalcon effectively maps entities and relations within a short text to its mentions of a background knowledge graph. It overcomes the challenges of short text using a light-weight linguistic approach based on a background knowledge graph.

NSSC has three configurable parameters (see Table 3) that must to be set appropriately. To achieve this, we present ablation studies described in Section 5.1, where we tune each module separately in a sequential search space. Since the term must be among the candidates selected by the LLM, the linking part must be optimized to present the correct candidates. Then, we compared NSSC with the parameters selected in the ablation study with the baselines in Section 5.2.

### Ablation studies

This section details the process of configuring various components of NSSC. The initial results related to the NER model are described in [21]. Subsequently, our focus shifts exclusively to the entity linking aspect. Ablation studies are performed to distinguish the collaborative and independent functionalities of medical entity linkage and medical disambiguation.

In systems with a constrained number of modules, each equipped with its unique configuration parameters, the parameter search process becomes crucial. In our specific context, where modules operate autonomously, we advocate a strategic approach to parameter search. The primary purpose of each module is to optimize the accuracy of the candidates it presents. In simpler terms, each module should return the most accurate and relevant candidates, prioritizing their ranking within the top selections. We address *RQ1* by presenting a comparative analysis in Table 7. To configure the entity linking module, we have tested five different querying methods, each with an increasingly wide range of search results:

1. *Exact*: This method is strictly exact match. Only results that exactly match the query terms in the knowledge base are returned.
2. *Basic_fuzzy*: This method uses basic fuzzy matching, allowing for slight variations in the query terms.
3. *Multi_match_SPA*: This query method represents the highest level of fuzziness and targets Spanish terms.
4. *Multi_match*: The same level of fuzziness is as *Multi_match_SPA*, but can also return terms from any language.
5. *Multi_match_boosted*: This query method can return results in any language and boosted terms related to cancer which are prioritized in the search results, that is, if the term cancer appears in the term candidate, it has a higher score.

The metrics used for comparison are the following:

- Accuracy@1 (%): This represents the accuracy in retrieving the best-fit term from the BK. For instance, the *Multi_match_boosted* method has an accuracy of 54.02%, indicating that in 54.02% of the cases, the top term retrieved was the correct one.
- Accuracy@15 (%): This measures the accuracy when retrieving a broader set of terms, up to the 15 best matches. The *Multi_match_boosted* method demonstrates an 77.64% accuracy rate for the top 15 terms, suggesting that while the single best term may not always be correct, expanding the search to the top 15 yields a much higher likelihood of retrieving the correct terms.
- Execution time (s): Indicates the time spent in this initial phase of term retrieval. The exact method is the fastest, taking only 1.72 s, while the *Multi_match_boosted* method is the slowest, taking 137.46 s. The time is calculated as the sum of the duration required to search all the terms in the benchmark.

The entity linking component, which is an integral part of the model’s output, includes a heuristic decision step. The effect of changing the threshold in the optimized module, as shown in Table 8, when combined with the BK response, reveals changes in performance.

**Table 8:** Finnetuning $\theta _{\text {{opt}}}$ over the entity linking module

$\theta _{\text {{opt}}}$	0.50	0.60	0.70	0.80	0.90	0.95	0.96	0.97	0.98
$\bar{\text {Accuracy@15}}$	0.7634	0.72992	0.7128	0.7251	0.7534	0.7764	**0**.**7810**	0.7799	0.7799

The range $0.50< \theta _{\text {{opt}}} < 0.98$ represents the desired similarity level between the searched term and the term in the vocabulary

In our benchmark, *Breast-Norm-Benchmark*, the optimal value for $\theta _{\text {{opt}}}$ is 0.96. This means that the LLM will only be used if all candidates have less than 96% similarity, or if there are candidates with more than 96% similarity and more than one CUI. Table 9 reports on the results observed by combining medical entity linking and disambiguation when playing prompt engineering over LLMs with symbolic reasoning against the background knowledge. The reported results aim to address *RQ2* and *RQ3*.

**Table 9:** Comparison of GPT-3.5 Turbo and GPT-4 performance with different prompt types over NSSC

EL + LLM	Prompt type	$$\bar{\text {Accuracy}}$$	#Tokens	Costs ($)
GPT-3.5 Turbo	Zero Shot	–	–	–
	Few Shot	0.4423	82,329	**0**.**12**
	CoT	0.7508	318,862	0.49
GPT-4	Zero Shot	–	–	–
	Few Shot	0.8512	289,538	8.80
	CoT	**0**.**8834**	350,144	11.46

The cost and tokens are calculated with OpenAI API

The results show that a well-designed prompt and a competent LLM can significantly improve the performance of the medical entity linking component. However, a poor application of LLMs together with inappropriate prompts can drastically reduce the performance of the approach and increase the execution time. This is because the nature of the text in clinical notes differs significantly from the terms in UMLS. Forcing disambiguation on an LLM with a high tendency to hallucinate is worse than just getting the CUIs provided by the medical entity linking component. The best parameters for our case study are $q_{\text {type}}$: *Multi_match_boosted*, $\theta _{\text {sim}}$: 0.96, $p_{\text {LLM}}$: CoT (GPT4). These parameters were used for the rest of the validation study.

### Main results

We present a comparative analysis of NSSC against scispaCy and BioFalcon, focusing specifically on its performance in terms of accuracy and execution time. The objective is to identify the most effective model for processing oncology clinical notes. The results are presented in Table 10. Accuracy@5 is chosen because no term in the benchmark has more than five possible CUIs.

**Table 10:** Comparison of accuracy and execution time for different systems

System	$\bar{\text {Accuracy@5}}$	Execution time (s)
scispaCy en_core_sci_sm	0.2884	$3.65 \pm 0.01$
scispaCy en_core_sci_lg	0.2756	$3.72 \pm 0.02$
scispaCy en_core_sci_scibert	0.3021	$12.98 \pm 0.05$
BioFalcon	0.5502	$154.33 \pm 0.71$
NSSC	**0**.**8834**	$2502.34 \pm 2.12$

Best results are highlighted in *bold*. This execution time represents how much it takes to normalize the full benchmark

The NSSC model clearly outperforms the baselines with an exemplary accuracy of 88.34%. This represents a considerable advance over the scispaCy models (en_core_sci_sm and en_core_sci_lg), which both hover around an accuracy of 28.84%, and the en_core_sci_scibert variant at 30.21%. BioFalcon, while superior to scispaCy, still falls short with an accuracy of 55.02%.

The execution time of the NSSC model, at 2502.34 s, is significantly higher than that of its competitors. In stark contrast, the scispaCy models demonstrate exceptional efficiency, with en_core_sci_sm and en_core_sci_lg models completing tasks in approximately 3.65 and 3.72 s, respectively, and the en_core_sci_scibert variant in 12.98 s. BioFalcon also reports an execution time of 154.33 s for the whole dataset.

## Discussion

The architecture of NSSC, as compared to other approaches in Table 1, is a cancer domain-specific model designed to link terms to a biomedical vocabulary such as UMLS. Since UMLS comprises various vocabularies, NSSC is agnostic to the specific vocabulary being used. Other approaches focus on specific tasks such as NER or EL, while NSSC presents a complete pipeline that transforms clinical note free text into various vocabulary concepts. Other methods, such as those proposed in [54, 68], use BERT embeddings to calculate the similarity between terms and entities. However, NSSC uses the BM25 algorithm as other approaches [19, 50], which is faster, more efficient, and does not require any training data. However, this approach to disambiguate candidates requires the use of LLMs, which involves crafting prompts and results in increased memory and processing time.

NSSC solves the oncology-medicine linking problem with the highest accuracy. However, the execution time increases by almost three orders of magnitude compared to the smallest version of scispaCy. Despite the higher computational cost, one of the NSSC strengths lies in its potential to generalize across a wide range of diseases, not just oncology. The adaptability of BERT-based NER with symbolic reasoning and LLM enables the framework to be extended to different medical domains. Training the model on diverse medical datasets allows it to fine-tune entity identification and linking for different diseases, enhancing its versatility in clinical text processing. This feature makes NSSC a valuable asset for advancing information extraction across the healthcare landscape.

The time complexity of the algorithm is higher than other approaches that do not use LLMs, because models like GPT-4 are significantly more expensive than smaller domain-specific models due to the large number of parameters, which are in the order of billions. However, the approach is optimized to avoid using the LLM in every case, making it more efficient. In addition, the use of the LLM allows the approach to be generalized to other domains.

NSSC uses contextual information as a critical input for understanding disease entities in clinical notes. Different diseases require different levels of context sensitivity; for example, oncology entities often require a nuanced understanding of dependencies, while simpler diseases may benefit from less detailed contextual analysis. Future enhancements should include disease-specific contextual knowledge, allowing the model to adjust its context sensitivity based on the unique characteristics of each disease. This extension will ensure that the approach remains tailored to the nuances of different medical conditions, optimizing performance across a range of diseases.

The use of advanced AI models in healthcare requires careful consideration of ethical implications, particularly patient privacy, consent, and the handling of sensitive medical data. As these models operate within clinical records, strict adherence to privacy standards and ethical guidelines is essential. Transparency in decision-making and ongoing collaboration with healthcare professionals are also critical to building trust in the technology and ensuring its ethical use in healthcare.

## Limitations

This paper points out some limitations, mainly in the results.

- The execution time of the NSSC model averages between 5 and 15 s. This timeframe is limited by the response time of the OpenAI API, which can vary. This problem can be solved if the LLMs are executed locally and do not depend on network overhead.
- The experiments were performed only on a benchmark of more than 400 terms, could escalate significantly if we were to extract all the terms from a real hospital database. Therefore, financial considerations should be taken into account.
- The use of an NER model before the transition to the linking phase may propagate errors from the initial phase to the subsequent phase. However, state-of-the-art models have remarkably high accuracy metrics that make potential errors manageable and adapt to account for them. In the end, the LLM is responsible for the final decision.

It is important to note that this execution time may make the model unsuitable for real-time applications. However, once a term is linked, there is no need for repetitive mapping, paving the way for the creation of a knowledge base that links terms with their corresponding context. This can be implemented with a cache database that stores the entity, label, and linked terms.

## Conclusions and future work

The proposed hybrid AI system, NSSC, outperforms existing approaches and thus represents a promising framework to advance the field of information extraction from clinical narratives. Generalization to other diseases, coupled with considerations of training costs and disease-specific contextual information, highlights the potential impact of NSSC on diverse healthcare applications.

Although the benefits of NSSC are significant, it is critical to recognize the computational costs associated with training such advanced models. Training BERT-based NER, reasoning over symbolic systems, and running LLMs typically requires significant computational resources and large annotated datasets. The cost of acquiring and preprocessing such datasets, coupled with the computational demands during training, can be challenging, especially in resource-constrained environments. Future research should focus on reducing training costs and optimizing model architectures for resource-constrained environments. These efforts will improve the accessibility and adoption of our framework. In addition, future research should prioritize improving disease-specific contextual adaptability. In this way, NSSC contributes to ongoing efforts to harness the power of AI to transform medical information extraction for improved patient care and medical research.

## Data Availability

The corpus used to validate the entity linking approach, *Breast-Norm-Benchmark*, is available at 10.5281/zenodo.11185980.

## Appendix A. Prompt costs

We use models gpt-3.5-turbo and gpt-4 though Microsoft Azure. To establish a connection with the OpenAI API, we utilize the Azure endpoint. The price of this API depends on the model, and there are different prices for the input tokens and the output tokens, as detailed in https://azure.microsoft.com/en-us/pricing/details/cognitive-services/openai-service/.

## Footnotes

1 https://www.who.int/news-room/fact-sheets/detail/cancer

2 https://www.nlm.nih.gov/research/umls

3 https://opensearch.org/

4 https://docs.python.org/3/library/difflib.html

5 https://openai.com/blog/openai-api

6 https://www.cancer.gov/

7 https://spacy.io/
